# Phage-mediated lysis increases growth rate of surviving bacterial cells

**DOI:** 10.1016/j.isci.2026.115899

**Published:** 2026-04-25

**Authors:** Emanuele Fara, Benjamin Raach, Alessio Cavallaro, Justus Fink, Divvya Ramesh, Yongzhao Guo, Victoria Orphan, Alex R. Hall, Gabriele Micali, Martin Ackermann, Olga T. Schubert

**Affiliations:** 1 Department of Environmental Microbiology, Eawag - Swiss Federal Institute of Aquatic Science and Technology, Dübendorf, Switzerland; 2 Institute of Biogeochemistry and Pollutant Dynamics, Department of Environmental Systems Science, ETH Zurich, Zurich, Switzerland; 3 PhD Program in Microbiology and Immunology, Life Science Zurich Graduate School, ETH Zurich and University of Zurich, Zurich, Switzerland; 4 Division of Geological and Planetary Sciences, California Institute of Technology, Pasadena, CA, USA; 5 Institute of Integrative Biology, Department of Environmental Systems Science, ETH Zurich, Zurich, Switzerland; 6 Istituto di Ricovero e Cura a Carattere Scientifico, Humanitas Research Hospital, Rozzano, Italy; 7 Laboratory of Microbial Systems Ecology, School of Architecture, Civil and Environmental Engineering, École Polytechnique Fédérale de Lausanne (EPFL), Lausanne, Switzerland

**Keywords:** Microbiology

## Abstract

Bacteriophage infection and lysis are typically associated with bacterial death, yet they also release cellular components that can be reutilized by surviving cells, particularly under nutrient limitation. To quantify the short-term growth consequences of this process, we established a controlled system of two *Escherichia coli* lysogenic strains, one harboring a wild-type prophage and the other a temperature-inducible prophage, enabling selective lysis of a defined fraction of the population. Following induction and lysis, the resulting biomass loss was smaller than expected, consistent with rapid recycling of released material and enhanced growth of surviving cells. A phenomenological mathematical model captures these dynamics and identifies a fast, transient increase in growth rate that accounts for the observed biomass compensation. Independent single-cell microfluidic measurements confirmed this boost in growth rate. Together, this work provides quantitative insight into short-term nutrient recycling and growth dynamics following phage-mediated lysis during active bacterial growth.

## Highlights

• Lysis of a bacterial subpopulation transiently increases biomass of surviving cells
• Model shows that growth gains depend on both recycling efficiency and recycling speed
• Single-cell growth increases when cells are exposed to lysate-derived supernatant

## Graphical abstract

**Figure:**
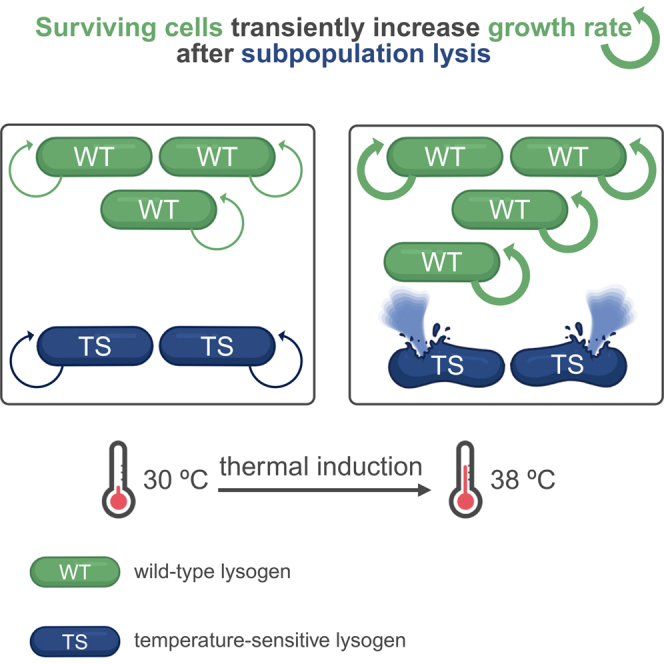

## Introduction

Bacteriophages, or phages, are viruses that infect bacteria and are widespread across diverse environments where their hosts occur, often at comparable or higher abundances than bacterial cells.^1^^,^^2^^,^^3^ Some phages can integrate their genetic material into the bacterial genome, forming prophages.^4^ These prophages can persist in their host across generations and are widespread among bacteria, with estimates suggesting that at least 20% and up to 60% of sequenced bacterial genomes contain one or more prophage elements.^5^^,^^6^^,^^7^^,^^8^ Prophages can be induced in response to various environmental and physiological triggers, initiating a lytic cycle that culminates in host cell death through lysis.^9^^,^^10^^,^^11^

Upon lysis, bacteria release both viral particles and a broad range of cellular components into the environment, such as amino acids, nucleotides, and vitamins.^12^^,^^13^ This process, known as the viral shunt, contributes to the recycling of dissolved organic matter within microbial ecosystems.^14^ In marine environments, for instance, it plays a key role in the cycling of carbon, nitrogen, and phosphorus by accelerating nutrient turnover and sustaining heterotrophic bacterial production.^15^^,^^16^^,^^17^^,^^18^ This process shapes bacterial community dynamics and influences how nutrients and energy flow through biogeochemical cycles.^19^

Despite the central role of the viral shunt in nutrient recycling across diverse ecosystems, its quantitative consequences for the short-term growth dynamics of surviving cells within a bacterial population remain less well resolved.^20^^,^^21^^,^^22^^,^^23^^,^^24^ Numerous studies have shown that lysis-derived material can support the growth of surviving cells within or across species under nutrient-limited conditions.^25^^,^^26^ Much of this work, however, has focused on cumulative biomass yield or long-term community effects,^27^^,^^28^^,^^29^^,^^30^ leaving less explored how lysis impacts the short-term growth dynamics of non-lysed cells during active growth. In addition, several experimental approaches rely on externally prepared lysates or bulk endpoint measurements, which do not resolve the timing and magnitude of growth-rate responses that can arise when phage lysis occurs in real-time within a population.^31^^,^^32^

The ecological consequences of biomass recycling depend strongly on the nutritional context in which phage-mediated lysis occurs.^33^ In nutrient-rich environments, the release of cellular components through lysis is unlikely to substantially alter the nutrient landscape and may have little impact on bacterial proliferation.^34^ In contrast, under nutrient-poor conditions, even modest nutrient inputs can significantly influence bacterial metabolism and transiently accelerate growth.^35^^,^^36^^,^^37^^,^^38^ In such contexts, phage-mediated lysis can represent an important source of accessible nutrients for non-lysed cells.^13^ Together, these observations suggest that, under resource-limited conditions, biomass loss due to lysis may be partially compensated by enhanced growth of surviving cells.

To quantify how prophage induction and subsequent lysis influence the short-term growth of non-lysed cells within the population, we combined batch culture experiments, mathematical modeling, and microfluidic growth assays.^39^ Batch cultures allowed us to monitor population-level dynamics under well-mixed conditions, where nutrients released from lysed cells rapidly diffuse and can be reutilized by non-lysed cells. To interpret these dynamics and assess how lysis affects growth rates, we developed a phenomenological mathematical model incorporating Monod-based growth kinetics and biomass recycling. The model enabled systematic exploration of parameter regimes consistent with the observed population-level responses. Finally, we confirmed our findings using microfluidics coupled with time-lapse microscopy. Together, these complementary approaches provide a quantitative framework to characterize how phage-mediated lysis transiently shapes bacterial population growth dynamics.

## Results

**Figure 1:**
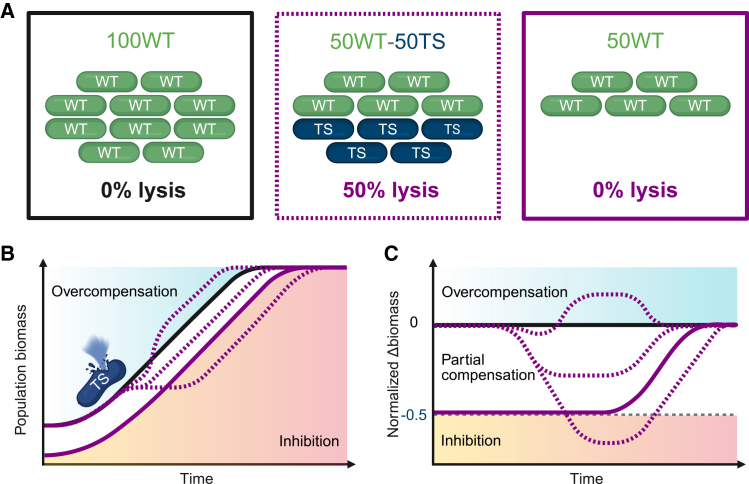
Conceptual overview of the co-culture system to assess phage-mediated lysis effects on bacterial growth dynamics (A) Schematic representation of initial population compositions. In this example, WT and TS lysogens are mixed at defined proportions, where 50% of the cells are TS lysogens that lyse upon prophage induction at 38°C (50WT–50TS, middle with purple dotted outline). A WT-only population with the same initial cell number serves as a control without lysis (100WT, left, with full black outline), while a WT-only population with the same initial number of WT cells as the mixed population provides a reference for growth comparison (50WT, right, with full purple outline). Note that this schematic is illustrative; actual experiments used different WT-TS proportions. (B) Expected growth dynamics of the WT-only and mixed populations, where prophage induction leads to the lysis of the TS lysogen subpopulation in the mixed population (purple dotted line). Subsequent growth of this mixed population can follow three scenarios: overcompensation, where biomass transiently exceeds that of the 100WT control (above the black line); inhibition, where biomass drops below that of the 50 WT population (below the purple line); or partial compensation, where WT cells recycle some nutrients released by lysis but do not fully restore the biomass of the 100 WT population. (C) Same scenarios as in B, but the vertical axis shows the biomass difference normalized to the 100WT control, calculated as (biomass-100WT biomass)/100WT biomass. This highlights the extent of biomass compensation or loss relative to the corresponding non-lysing population.

To investigate how phage lysis influences the short-term biomass and growth dynamics of non-lysed cells within a bacterial population, we established an experimental system using two lysogenic strains derived from *Escherichia coli*. One strain carries a wild-type (WT) lambda prophage (“WT lysogen”) stably integrated into the genome and is immune to superinfection by lambda phage particles. The other strain harbors a temperature-sensitive (TS) lambda variant (“TS lysogen”), which remains dormant at 30°C but is induced into the lytic cycle at 38°C.^40^^,^^41^^,^^42^ To distinguish the two strains, each was engineered to constitutively express a fluorescent reporter: the WT lysogen expresses a green fluorescent protein (GFP), while the TS lysogen expresses a red fluorescent protein (RFP). Combining the two strains in defined proportions enables precise control over the fraction of cells in which phage-mediated lysis is selectively induced. In our workflow, each strain was precultured at 30°C to prevent induction of the TS lysogen, after which the two strains were mixed, and the resulting culture was shifted to 38°C for all main experiments (Figure 1A).

Thermal induction of the TS lysogen results in extensive lysis of the TS subpopulation,^42^ accompanied by the release of cellular components that can serve as a nutrient source for the surviving WT cells. In nutrient-poor conditions with glucose (0.1%) as the sole externally supplied carbon source, as in our experiments, the population-level outcome depends on the balance between the loss of a fraction of cells and the benefit gained from the released nutrients. Three scenarios can be envisioned based on how the population responds to the lysis of a subpopulation (Figure 1B). In an overcompensation scenario, components released by lysed cells increase growth in surviving cells to the extent that it results in transiently higher biomass than in populations without lysis^43^; this could be expected if some of the released components act as signals that induce increased cell growth.^44^ In contrast, inhibition occurs when the release of cellular components has a detrimental effect, such as signaling stress or damage that slows or halts the growth of surviving cells, leading to an even bigger loss in biomass than what is directly caused by phage-mediated lysis.^25^^,^^45^^,^^46^ Between these extremes lies a continuum of intermediate outcomes in which the biomass loss is either fully compensated by increased biomass production thanks to the released nutrients, partially compensated, or remains uncompensated.^47^ These scenarios provide a framework to interpret the population dynamics observed across varying proportions of lysing cells.

To visualize the effect of lysing cells on the population growth, we computed the relative difference in biomass compared to a non-lysing control over time. This “normalized Δbiomass” metric provides a clear criterion to evaluate whether lysis results in overcompensation, partial compensation, or inhibition (Figure 1C).

### Surviving cells partially compensate for biomass loss from phage-mediated lysis

To quantify the effect of phage-mediated lysis in batch cultures, we prepared mixed populations by combining WT and TS lysogens at defined proportions (100WT, 73WT–27TS, 19WT–81TS, and 100TS), each adjusted to an initial optical density (OD) at 600 nm (OD_600_) of 0.01. We also included control populations with only WT lysogens at reduced initial OD_600_ (73WT and 19WT), matching the WT fractions in the mixed populations but without any TS cells. The cultures were incubated at 38°C to induce lysis in the TS subpopulation. Total population biomass was monitored over time using OD_600_ measurements, while the biomass of WT lysogens was specifically quantified by GFP-based cell counts via flow cytometry.

**Figure 2:**
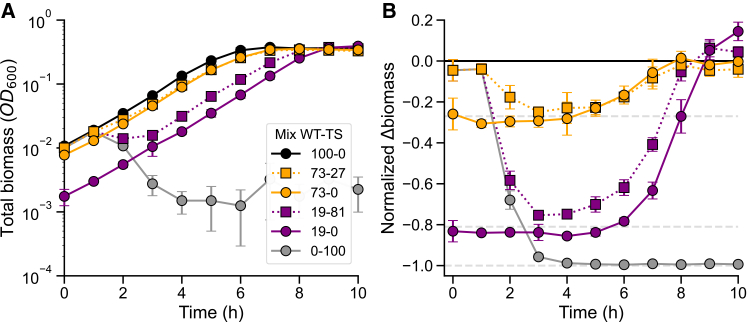
Bacterial biomass loss from phage-mediated lysis is partially compensated for by non-lysed cells (A) Growth curves showing total biomass over time for bacterial populations composed of WT and TS lysogens mixed at the indicated proportions and grown at 38°C, a temperature that induces phage-mediated lysis of TS cells. The 100WT, 73WT, and 19WT populations (WT only, full lines) exhibit steady exponential growth but start at different initial OD_600_, reflecting the decreasing initial fraction of WT cells; this establishes a baseline for comparison with the mixed WT-TS populations (73WT-27TS and 19WT–81TS, dotted lines), where part of the biomass is lost to phage-mediated lysis. Each data point represents the mean ± standard deviation (SD) from four biological replicates. See also Figure S1 for WT-only biomass measurements. (B) Growth curves normalized to the 100WT control. Here, it is well visible that the 19WT-81TS population exhibits partial compensation, as its biomass never decreases to the level of the corresponding control (19WT) until saturation, suggesting that the biomass loss of TS lysogens is partially compensated. In the case of the 73WT-27TS population, the trajectory more closely resembles that of its control (73WT), indicating that the absolute effect is less pronounced at lower TS fractions.

As expected, the WT-only populations (100WT, 73WT, and 19WT) exhibited steady exponential growth, while the TS-only population (100TS) initially followed the same trajectory but showed a sharp decline in biomass due to extensive lysis after 1 h (Figure 2A). In the presence of both the WT and the TS lysogens (73WT–27TS and 19WT–81TS), the mixed populations initially grew similarly to the 100 WT control until the TS fraction began to lyse after about 1 h. This lysis event caused a decline in biomass proportional to the fraction of TS cells, with the most pronounced drop observed in the 19WT–81TS mixture (Figure 2B). Importantly, the biomass of the two mixed populations did not drop to the level of their non-lysing controls with a lower initial OD_600_ (73WT and 19WT). The 19WT–81TS population consistently exhibited higher biomass than its non-lysing control (19WT) until saturation, indicating partial compensation for biomass lost through lysis. By contrast, the 73WT–27TS mixture closely followed the trajectory of its corresponding control (73WT), suggesting that a substantial fraction of lysis is needed to produce a clearly detectable impact on the surviving WT population.

WT cell count measurements confirmed that the 19WT–81TS population maintained higher WT cell numbers than the 19WT control throughout the time course, despite starting with the same initial number of WT cells (Figure S1). This increase occurred after TS cell lysis, indicating that the total biomass gain in mixed populations results from a net increase in WT biomass. The high specificity of the flow cytometry-based quantification for GFP-expressing WT cells over RFP-expressing TS cells rules out the possibility that residual, non-lysed TS cells substantially contributed to the observed increase in total biomass measured via OD_600_ (Figures S1A and S1B). Consistently, bulk measurements of GFP fluorescence in the cultures yielded results nearly identical to the flow cytometry data, further supporting this interpretation (Figures S1C and S1D). In contrast, the 73WT–27TS population showed no detectable increase in cell counts or bulk GFP fluorescence compared to its control, aligning with the absence of a significant effect in OD_600_ measurements.

### A mathematical model captures how increased growth rate drives partial biomass compensation

**Figure 3:**
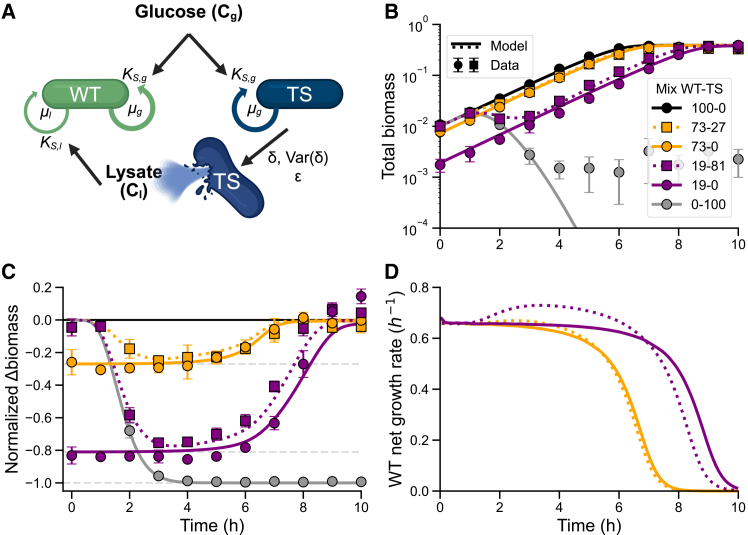
A mathematical model recapitulates partial biomass compensation driven by transient growth rate increase of surviving cells (A) Schematic of the consumer-resource model showing WT and TS lysogens growing on glucose (C_g_), a shared resource, with a Monod constant *K*_*S,g*_ and a growth rate *μ*_*g*_. TS lysogens lyse at rate *δ* and Var(*δ*), releasing lysate (C_l_) that can be consumed by both WT and TS cells as an additional resource, with lysate-dependent growth rate *μ*_*l*_, half-saturation constant *K*_*S,l*_, and biomass recycling efficiency *ε*. See also Figure S2 for the parameter sensitivity analysis and Table S1 for the best-fit model parameters. (B) Simulated growth dynamics for populations with varying WT-TS proportions. Experimental data (data points represented as mean ± SD, same as in Figure 2A) are overlaid on model simulations (lines) to illustrate the agreement across populations with different WT-TS proportions. (C) Same data as in (B), but biomass values are normalized to the 100WT control to highlight partial biomass compensation. (D) Simulated net growth rate of WT cells in the 19WT-81TS mixed population (dotted line) compared to the 19WT monoculture control (solid line). The mixed population exhibits a transient increase in WT growth rate between 1 and 7 h, corresponding to the period of TS subpopulation lysis, with a peak around 3 h. See also Figure S3 for the dynamics of TS lysis.

Building on our experimental observation that mixed populations with 81% lysing cells showed only a partial drop in total biomass that is less than expected given the fraction of lysed cells, we hypothesized that the surviving WT subpopulation compensates for the loss through an increased growth rate. To assess whether an increased growth rate of surviving cells could account for the observed population-level dynamics, we developed a mathematical model focused on identifying the parameters that most strongly influence the growth of non-lysed cells (Figure 3A). The model builds on a consumer-resource framework^48^ with two bacterial species as in the experimental system, the WT and TS lysogens. Both lysogens grow on glucose (C_g_), described by Monod kinetics with a maximum growth rate (*μ*_*g*_) and a half-saturation constant (*K*_*S,g*_). TS lysogens lyse at a rate (*δ*), releasing lysate (C_l_) that can be consumed by both WT and TS cells as an additional nutrient source. Because TS cells are progressively removed following induction, lysate-derived growth contributes primarily to the WT population, with only minor effects from transient uptake by TS cells. To capture the single-cell heterogeneity of the induction to lysis time,^49^^,^^50^ the model distributes the TS biomass across sequential compartments, enabling control over the variance in lysis timing (Var(*δ*)) by adjusting the number of compartments. WT lysogens consume lysate governed by Monod kinetics with a maximum growth rate (*μ*_*L*_) and a half-saturation constant (*K*_*S,l*_). For simplicity, and to limit the number of unconstrained parameters, we model the lysate as a single effective resource pool where *K*_*S,l*_ serves as an aggregate half-saturation constant although the lysate likely consists of multiple components with distinct uptake kinetics. The contribution of this lysate-based growth is scaled by a biomass recycling efficiency parameter (*ε*), which defines the fraction of lysed biomass converted into new biomass and can be interpreted as the yield on lysate.

We parametrized the model in three sequential stages to reflect the biological processes at play (see STAR Methods and Table S1). The resulting model reproduces the observed dynamics within the explored parameter regime: biomass declines following lysis but partially recovers over time as WT cells regrow on released lysate, capturing the transient growth boost underlying partial compensation (Figures 3B and 3C). We also performed a parameter sensitivity analysis using a systematic parameter sweep, which illustrates how the main qualitative features of the simulated dynamics respond to variations in the key parameters (Figure S2).

To directly evaluate whether increased WT growth could account for the partial biomass compensation observed experimentally, we examined the model-derived WT subpopulation growth rate (Figure 3D). In the 19WT monoculture, the growth rate remains approximately constant while cells grow on glucose. By contrast, in the mixed 19WT–81TS population, the model parameterized with experimental data shows a pronounced transient increase in WT growth rate between 1 and 7 h, with a peak at around 3 h. At its maximum, this increase corresponds to an approximately 11% higher WT growth rate compared to the control. This early, transient growth rate increase is consistent with rapid uptake of released nutrients by surviving WT cells, as lysis begins at approximately 1 h and WT growth peaks shortly thereafter in the simulation (Figure S3). Together, these dynamics account for the partial biomass compensation observed in the experiments.

### Exposure to phage-mediated bacterial lysate increases single-cell growth rates

To test whether the partial compensation we observed in batch culture could be accounted for by a transient increase in growth rate as the model suggests, we used a microfluidic mother machine device coupled with fluorescence microscopy to capture time-lapse images of individual cells growing under defined conditions.^39^ These images were analyzed using an automated image analysis pipeline to extract cell elongation over time, enabling accurate quantification of single-cell growth rates. By maintaining constant environmental conditions, our microfluidic setup minimizes confounding factors inherent to batch cultures, such as nutrient depletion, fluctuating cell densities, and asynchronous lysis events.

**Figure 4:**
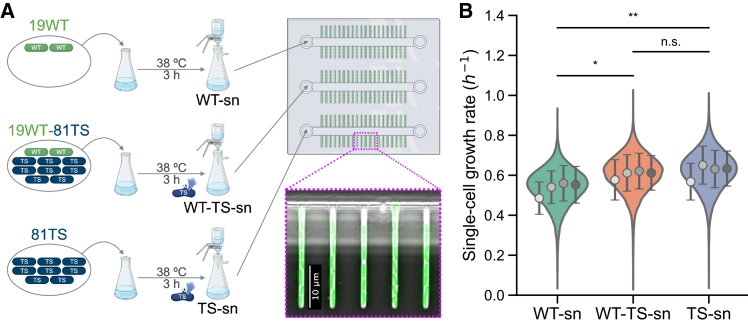
Phage-mediated bacterial lysate increases single-cell growth rates (A) Schematic of the supernatant preparation and the experimental setup for single-cell growth measurements using a mother machine microfluidic device and time-lapse fluorescence microscopy. Three types of culture supernatants were prepared by culturing individual or mixed populations of WT and TS lysogens at 38°C for 3 h: WT-sn (WT-only control), WT-TS-sn (WT and TS mix, with extensive lysis), and TS-sn (TS-only lysate). After filtration to remove cells, each supernatant was flowed through the microfluidic device loaded with WT lysogens. The layout of the device is shown schematically, with a zoom-in displaying a representative fluorescence microscopy image. See also Video S1 for an example of mother machine data and Figure S4 for the estimation of lysed-derived biomass and the quantification of supernatant properties. The scale bars represents 10 μm. (B) Single-cell growth rate distributions of WT lysogens exposed to the three different culture supernatants. Cells exposed to lysate-containing supernatants, WT-TS-sn and TS-sn, showed significantly higher growth rates compared to the WT-sn control (*p* = 0.024 and *p* = 0.008, respectively), while no significant difference was observed between the two lysate conditions (*p* = 0.764). For the condition WT-sn, a total of *n* = 10*,*221 cells were analyzed; for WT-TS-sn, *n* = 10*,*878; and for TS-sn, *n* = 8*,*992. Mean growth rates and standard deviation were 0.54 ± 0.09 h^−1^ for WT-sn, 0.61 ± 0.09 h^−1^ for WT-TS-sn, and 0.62 ± 0.10 h^−1^ for TS-sn. Violin plots represent pooled single-cell measurements from four independent biological replicates. Means and standard deviations for each replicate are overlaid. Statistical analysis was performed on the means of biological replicates using a two-way ANOVA, with condition and experiment (biological replicate) as factors, followed by Tukey’s post hoc test. Additional methodological details are provided in the STAR Methods, Tables S2, S3, and S4.

In preparation for these experiments, we collected three cell-free supernatants from batch cultures designed to match the conditions of previous experiments (Figure 4A). Each culture was inoculated with WT-TS mixtures at an initial OD_600_ of 0.01: (1) WT-supernatant (sn), a non-lysing control containing only WT cells (19% of the initial OD_600_; “sn” stands for supernatant); (2) WT-TS-sn, a mixed culture with 19% WT and 81% TS cells; and (3) TS-sn, a pure TS culture (81% of the initial OD_600_). Lysis was induced by incubating cultures at 38°C, and supernatants were harvested after 3 h, when lysis was largely complete in TS-containing populations (Figure S4). These supernatants were then supplied to a microfluidic device preloaded with WT cells, which were subsequently imaged at 38°C to obtain single-cell growth rates.

The analysis of single-cell growth rates across the three conditions revealed clear and consistent differences (Figure 4B). WT lysogens exposed to the 81% TS lysate (TS-sn) showed the highest mean growth rate of 0.62 ± 0.10 h^−1^ (mean ± standard deviation), followed closely by those exposed to the WT-TS-sn (0.61 ± 0.09 h^−1^). In contrast, cells grown in the control supernatant from the 19% WT-only culture (WT-sn) grew significantly more slowly, with a mean growth rate of 0.54 ± 0.09 h^−1^ (ANOVA + Tukey’s honestly significant difference (HSD): *p* = 0.008 for TS-sn vs. WT-sn; *p* = 0.024 for WT–TS-sn vs. WT-sn). This represents a ∼13%–15% increase in growth rate when WT lysogens are exposed to lysate-containing supernatants, closely matching the growth-rate increase estimated by the model (Figure 3D). When translated into doubling times, this corresponds to an acceleration of nearly 9 min per cell cycle, from ∼77 min in the control (WT-sn) to ∼68 min in the lysate conditions.

Together, these single-cell experiments provide direct evidence that exposure to phage lysate increases the growth rate of surviving cells. This enhancement occurred despite the relatively small amount of lysate present, estimated to correspond to a biomass loss of approximately 2 × 10^4^ cells/mL (based on flow cytometry measurements, Figures S4A and S4B). This loss is minor compared to the final biomass of a culture in the stationary phase, where cell densities are at least three to four orders of magnitude higher. Importantly, the batch cultures from which the supernatants were collected maintained baseline pH and glucose levels at the time of sampling (Figures S4C and S4D), indicating that the observed effect on growth rate was not affected by acidification or nutrient depletion. Instead, the data support a direct nutritional effect of lysis-derived components, consistent with the model indicating that even limited lysate availability can transiently accelerate growth in surviving cells.

## Discussion

The amount of biomass released globally through viral lysis is enormous. Most estimates come from marine systems, where viruses kill 15%–40% of bacteria each day,^51^ and studies in other environments report values in a similar but more variable range. Even under conservative assumptions of only 0.5%–4% daily mortality,^52^^,^^53^^,^^54^ the resulting biomass release is still comparable to the estimated global biomass of all animals.^55^ Understanding how this released biomass influences the physiology and growth of other microbes is therefore an important goal.

Our results demonstrate that phage-mediated lysis, beyond its role in cell death and viral propagation, can directly influence the short-term growth dynamics of surviving cells. Using a controlled mixture of inducible and non-inducible lysogens, we show that lysis leads to a transient but significant increase in the growth rate of the surviving population, supporting the idea that nutrients released upon lysis are rapidly taken up and metabolized by non-lysed cells. This growth acceleration results in partial compensation of the biomass lost due to cell death, revealing a direct and dynamic link between lysis and growth at the population level. Rather than serving solely as a mechanism of passive biomass recycling, our findings demonstrate that lysis can act as an immediate source of growth-promoting resources that partially buffer biomass loss under nutrient-poor conditions. In contrast to the previously described role of cell lysis in nutrient recycling during stationary phase,^56^ phage infection and lysis predominantly target actively growing cells. This study thus offers quantitative insights into how lysis-driven nutrient turnover can influence populations during active growth.

One plausible mechanism involves uptake of lysate-derived metabolites that alleviate metabolic constraints by providing readily accessible resources.^57^^,^^58^^,^^59^ Key cellular building blocks such as amino acids, nucleotides, and carbohydrates are released upon lysis and can be directly assimilated, minimizing the demand for costly biosynthesis and accelerating biomass production.^13^^,^^60^^,^^61^^,^^62^ Additionally, released cofactors and vitamins may stimulate enzymatic activity,^63^ further increasing the cells’ metabolic capacity. Lysate may also contain signaling molecules or regulatory factors that influence gene expression and redirect metabolism toward specific functions.^64^ Together, these elements can support faster growth in surviving cells, particularly under nutrient-limited conditions. Future work will be needed to quantify the effects of individual lysate components and how they may be integrated into new biomass. Such efforts would help elucidate the specific regulatory and metabolic pathways activated in surviving cells that have access to lysate.

Overall, we provide quantitative evidence that phage-mediated lysis, beyond imposing a cost through cell loss, can serve as a transient source of nutrients that enhances the growth rate of surviving cells in a well-mixed system. Beyond our synthetic system, these dynamics may also be relevant in natural bacterial communities, especially in nutrient-limited environments where even modest resource pulses can influence bacterial activity.^65^^,^^66^ A timely next step would be to investigate how these dynamics unfold in spatially structured systems, which are common in nature, such as marine particles or biofilms, where lysis may not only locally enhance the growth rate of neighboring cells but also contribute to the formation and maintenance of these structures.^67^^,^^68^ In addition, studying the long-term effects of lysis-driven resource release on bacterial populations, beyond the short-term effects quantified in this study, will provide further insights into its ecological role. Ultimately, our results reveal how prophage-mediated lysis can transiently reshape bacterial growth through rapid nutrient recycling, providing a quantitative framework for understanding how viral mortality and resource recycling jointly structure bacterial populations.

### Limitations of the study

Our experiments rely on a simple *Escherichia coli*-lambda phage model in well-mixed laboratory environments, capturing only a small fraction of the host-phage diversity and ecological complexity found in nature, and focusing on short-term growth responses under controlled conditions. Beyond differences in lysate composition arising from host or phage taxonomy and physiology, multispecies interactions, spatial structure, and environmental physical or chemical properties may further influence how lysis-derived material is distributed, accessed, and utilized. In addition, while prophage induction produces infectious phage particles, our system uses lysogenic strains with superinfection immunity, preventing secondary infection cycles. As a result, the study isolates the effect of lysis-derived nutrient release without capturing the ecological feedback between phage replication, host density, and resource availability that would occur in natural systems. The single-cell assays use supernatants rather than inducing lysis in real time, which provides precise control over exposure but does not resolve the immediate onset of responses following lysis; integrating real-time induction into microfluidic workflows would allow finer temporal characterization. Lysates generated during prophage induction may also contain lytic enzymes or other cellular components that could affect non-lysed cells in ways not examined here, meaning additional biochemical or signaling effects cannot be excluded. Finally, the impact of lysis-derived material may differ in nutrient-replete environments, under maximal growth, or during severe starvation, where additional physiological responses may emerge.

## Resource availability

### Lead contact

Requests for further information and resources should be directed to and will be fulfilled by the lead contact, Martin Ackermann (martin.ackermann@eawag.ch).

### Materials availability

• Bacterial strains generated in this study are available from the lead contact without restriction.
• The plasmid sequences generated in this study have been deposited in GenBank under accession numbers PV021963 and PV936258, and the plasmids are available from the lead contact upon request.

### Data and code availability

• All the raw imaging data generated in this study have been deposited in the Eawag Research Data Institutional Collection (ERIC) and are publicly available as of the date of publication at https://doi.org/10.25678/000GGA.
• All original code has also been deposited in the Eawag Research Data Institutional Collection (ERIC) and is publicly available as of the date of publication at https://doi.org/10.25678/000GGA.
• Any other information required to reanalyze the data reported in the article is available from the lead contact upon request.

## Acknowledgments

We thank Ido Golding for providing the temperature-sensitive and wild-type lambda phages, Carolin Wendling for teaching phage techniques, Rachel E. Szabo and Simon van Vliet for valuable discussions, and Kim Schlegel for technical support. We also thank Frederik Hammes for his valuable contribution during experimental troubleshooting. Furthermore, we are grateful to the current and former members of the Microbial Systems Ecology lab for their constructive feedback, to Thierry Emonet for his guidance, and to Franziska Oschmann, Janis Fluri, Thomas Wuest, and Lukas von Ziegler for developing the MIDAP pipeline used for automated image analysis. This research was supported by 10.13039/501100003006ETH Zurich and Eawag, the 10.13039/501100011718Swiss Federal Institute of Aquatic Science and Technology. Additionally, it was funded as part of the 10.13039/501100023650NCCR Microbiomes, supported by the Swiss National Science Foundation (grant numbers 51*NF*40 180575 and 51*NF*40 225148 to M.A. and O.T.S.), and through a project grant from the Swiss National Science Foundation (188642 to M.A.). The graphical abstract and Figures 1, 3A, 4A, S2A, and S5 were created using BioRender.com.

## Author contributions

Conceptualization, E.F., G.M., M.A., and O.T.S.; data curation and formal analysis, E.F. and B.R.; methodology, E.F., B.R., J.F., A.C., Y.G., and V.O.; investigation, E.F., B.R., A.C., and D.R.; project administration, E.F.; funding acquisition, M.A. and O.T.S.; supervision, A.R.H., M.A., and O.T.S.; writing – original draft, E.F.; writing – review and editing, E.F., B.R., O.T.S., M.A., and all other co-authors.

## Declaration of interests

The authors declare no competing interests.

## Declaration of generative AI and AI-assisted technologies in the writing process

During the preparation of this work, the authors used ChatGPT (v.4o, 5, 5.1, and 5.2) to assist with text rephrasing, code debugging for data analysis, and data organization prior to submission. All outputs generated by the tool were subsequently reviewed and edited by the authors, who take full responsibility for the final content of the publication.

## STAR★Methods

### Key resources table

**Table:** 

REAGENT or RESOURCE	SOURCE	IDENTIFIER
**Bacterial and virus strains**		

WT lysogen	This study; *Escherichia coli* BW25113 from the Keio collection (Baba et al.^69^) with prophage lambda and plasmid pEF001	N/A
TS lysogen	This study; *Escherichia coli* BW25113 from the Keio collection (Baba et al.^69^) with temperature-sensitive prophage lambda and plasmid pEF002	N/A
Phage lambda	Zong et al.^42^	λ_IG831_
Temperature-sensitive phage lambda	Zong et al.^42^	λ_IG2903_

**Chemicals, peptides, and recombinant proteins**		

Luria Bertani Broth with agar (Lennox)	Sigma-Aldrich	L2897
M9, Minimal Salts, 5x	Sigma-Aldrich	M6030
Kanamycin	Sigma-Aldrich	K1876
Chloramphenicol	AppliChem	A1806
Glucose	Sigma-Aldrich	G8270

**Deposited data**		

Raw and analyzed data	This study; ERIC: [2026]	https://doi.org/10.25678/000GGA
Analysis scripts	This study; ERIC: [2026]	https://doi.org/10.25678/000GGA

**Oligonucleotides**		

DNA fragments for plasmids pEF001 and pEF002	This study; see Table S5	N/A
Primers for plasmids pEF001 and pEF002	This study; see Table S6	N/A

**Recombinant DNA**		

Plasmid pEF001	This study; see Figure S5A	GenBank: PV021963
Plasmid pEF002	This study; see Figure S5B	GenBank: PV936258

**Software and algorithms**		

Python 3.10.4	Python Software Foundation	https://www.python.org/downloads/release/python-31017/
MIDAP 0.3.18	Developed in-house by the Microbial Systems Ecology Group at Eawag and ETH Zurich in collaboration with ETH Scientific IT Services	https://github.com/Microbial-Systems-Ecology/midap.git
Omnipose algorithm (Version 0.4.4)	Cutler et al.^70^	https://github.com/kevinjohncutler/omnipose.git
STrack algorithm (Version 4)	Todorov et al.^71^	https://github.com/Helena-todd/STrack.git
Benchling	Benchling^72^	RRID: SCR_013955

### Experimental model and study participant details

#### Bacterial strains

Two lysogenic derivatives were constructed by infecting *Escherichia coli* BW25113^69^ with either wild-type lambda phage or a temperature-sensitive variant carrying the thermolabile cI857 repressor allele.^40^ These strains are referred to as the WT lysogen and TS lysogen, respectively. Both phages contain a kanamycin-resistance cassette for stable maintenance. The WT and TS lysogens were transformed with plasmids pEF001 and pEF002, respectively: pEF001 drives constitutive expression of a green fluorescent protein in the WT lysogen, while pEF002 enables constitutive expression of a red fluorescent protein in the TS lysogen.

#### Plasmid construction

To label and distinguish the two lysogenic strains, we constructed two dual-reporter plasmids from scratch. Each plasmid encodes two fluorescent proteins: one expressed during the lysogenic state and the other upon induction of the lytic cycle. The choice of fluorescent proteins was guided by compatibility in excitation/emission spectra and brightness, using information from the FPbase database.^73^ The reporters were placed in a divergently transcribed arrangement, separated by the lambda *O*_*R*_ operator region, which includes binding sites *O*_*R*_*1*, *O*_*R*_*2*, and *O*_*R*_*3* for the cI repressor.

The plasmid pEF001 (Figure S5A) expresses the green fluorescent protein *sfGFP* (hereafter GFP) during the lysogenic cycle and switches to expressing the cyan fluorescent protein *mCerulean* (CFP) upon lytic induction. Similarly, pEF002 (Figure S5B) expresses the red fluorescent protein *mCherry2* (RFP) during lysogeny and transitions to yellow fluorescent protein *mVenus* (YFP) during lysis. Notably, while the transcription of the lysogenic reporter ceases upon lytic induction, the fluorescent protein remains in the cell until it is degraded.

To enhance turnover of the lysogenic reporter and improve signal specificity during the transition to the lytic state, both plasmids include the *mf-lon* gene encoding the Lon protease. This protease selectively degrades proteins tagged with a specific degradation sequence. Based on a published study,^74^ the *pdt#3* tag – a 23 amino acid sequence – was fused to the N-terminus of the lysogenic reporters (GFP in pEF001 and RFP in pEF002), rendering them substrates for Lon-mediated degradation. To prevent premature degradation during lysogeny, the *mf-lon* gene was positioned downstream of the lytic reporter, ensuring that Lon is only expressed upon entry into the lytic cycle. Both plasmids were built on a p15A origin of replication, yielding a medium copy number (20–30 copies per cell),^75^ and carry a chloramphenicol resistance cassette (*CAT/cmR*) for plasmid maintenance.

Plasmid sequences were designed *in silico* using Benchling and synthesized as five DNA fragments (gBlocks HiFi Gene Fragments, Integrated DNA Technologies; see Table S5). These fragments were mixed in equimolar ratios and assembled using the Gibson assembly method,^76^ with the NEBuilder HiFi DNA Assembly Master Mix (NEB #E2611), following the manufacturer’s instructions. Assembled constructs were transformed into chemically competent *E. coli* and plated on LB agar with 25 μg/mL chloramphenicol. Colonies were screened via colony PCR, and one clone per plasmid was selected and verified by Sanger sequencing using primers listed in Table S6. The sequences of pEF001 and pEF002 were deposited in GenBank under accession numbers PV021963 and PV936258, respectively.

Plasmids were extracted from *E. coli* competent cells (NEB #C2987H) using the QIAprep Spin Miniprep Kit (Qiagen #27104) and transformed into the WT and TS lysogenic strains. The resulting strains express GFP (WT lysogen) or RFP (TS lysogen) during the lysogenic state. Upon induction of the lytic cycle, the TS lysogen switches to YFP expression and degrades the RFP reporter, allowing dynamic tracking of prophage activation.

### Method details

#### Batch culture experiments

The WT and TS lysogens were streaked from 25% glycerol stocks onto plates with Luria Bertani Broth with agar (Lennox, Sigma-Aldrich L2897) supplemented with 50 μg/mL kanamycin (Sigma-Aldrich K1876) and 20 μg/mL chloramphenicol (AppliChem A1806) to maintain the prophage and the reporter plasmid, respectively. The plates were incubated at 30 °C overnight (approximately 16 h) and then kept in the fridge at 4 °C. From the Petri dishes, single colonies were inoculated in culture tubes with 3 mL of M9 minimal medium (Sigma-Aldrich M6030) supplemented with 0.1% (w/v) glucose (Sigma-Aldrich G8270), 50 μg/mL kanamycin, and 20 μg/mL chloramphenicol, and incubated at 30 °C with 250 rpm shaking for approximately 24 h to reach stationary phase. These cultures were then diluted 1:100 into flasks with 20 mL of M9 minimal medium plus the above-mentioned supplements and incubated at 30 °C for approximately 16 h to reach the end of the exponential phase. The resulting cultures were then used for the batch culture experiments.

For the batch culture experiments, the two lysogens were mixed in various proportions raging from 100WT, 91WT–9TS, 73WT–27TS, 19WT–81TS, and 100TS in a flask with 100 mL of M9 minimal medium plus the above-mentioned supplements with a total starting OD_600_ of 0.01. The flasks were placed in a water bath (Grant OLS Aqua Pro) set to 38 °C with 140 rpm shaking to induce lysis of the TS lysogens.

#### Preparation of lysate-containing supernatants

The media for the microfluidic experiments were prepared following the same protocol as for the batch culture experiments, with the WT–TS proportions of 19WT, 19WT–81TS, and 81TS. The cultures were stopped after 3 h, and the supernatants were collected after two sequential filtrations using PES filter membrane with pore size of 0.22 μm (TPP Filtermax, Sigma Z760897). Each supernatant was tested for sterility by incubating 3-mL cultures at 37 °C overnight and checking the OD_600_. In addition, we measured the pH (FiveEasy Plus FP20 pH/mV Meter, Mettler Toledo) and glucose concentration (Glucose Colorimetric Detection Kit, Invitrogen EIAGLUC) to ensure that the medium was not abnormally acidic and that glucose was not substantially depleted (Figure S4).

#### Microfluidic device fabrication

The microfluidic device (wafer W001 from the group’s database) consisted of a main channel with a height of 20 μm and width of 50 μm, flanked by side chambers measuring 25 μm (length) × 1.1 μm (width) × 1 μm (height). Devices were fabricated by replica molding polydimethylsiloxane (PDMS; Sylgard 184, Dow Corning), mixed at a 10:1 elastomer-to-curing-agent ratio, onto a silicon wafer master mold. The mixture was cured at 80 °C for 2 h, then cut into chips of approximately 3.5 cm × 3.5 cm. Inlet and outlet holes (0.5 mm diameter) were manually added using a hole puncher (Welltech Rapid-Core). The chips were bonded to 50-mm diameter glass cover slips (Menzel-Gläser) by plasma activation (30 s at maximum power; Plasma Cleaner PDC-32G-2, Harrick Plasma), followed by contact bonding and heating on a 100 °C hot plate for 1 min. All chips were assembled and bonded on the day of the experiment.

#### Microfluidic experiments using time-lapse microscopy

We broadly followed the microfluidic procedure described previously by Micali and colleagues.^77^^,^^78^ Briefly, after loading cells into the chip, media were delivered via syringe pumps (NE-300, NewEra Pump Systems) using 50-mL syringes containing one of the three supernatants. Syringes were connected to the device using a 20-G needle (0.9 mm × 70 mm) attached to Microbore tubing (Saint-Gobain Tygon Non-DEHP, ID 0.76 mm, OD 2.29 mm, thickness 0.76 mm; Fisher Scientific) cut to ∼8 cm. A narrower PTFE tubing (ID 0.3 mm, OD 0.8 mm; Adtech, 39172900) cut to ∼60 cm was connected at the other end and inserted into the PDMS chip inlets. Cells were first loaded into the lateral chambers using small air bubbles to assist entry through the main channel. Fresh medium was then perfused through the main channel, and cells were allowed to acclimate for ∼2 h. Time-lapse imaging was performed over 12 h using an Olympus IX81 inverted microscope equipped with a 100× NA1.3 oil objective, an ORCA-Flash 4.0 v2 sCMOS camera, and an X-Cite120 lamp with Chroma 49000 series GFP filters. Autofocus was maintained via the Olympus Z-drift compensation system, and image acquisition was controlled by Olympus cellSens software. Imaging was conducted at 38 °C with multiple fields of view per device, captured every 5 min in GFP and phase contrast channels. Images were saved in *.ets* format and converted to *.tiff* using Fiji (ImageJ2 v2.14.0/1.54p) for analysis. Only the final 6 h of each experiment were analyzed. Five independent biological replicates were performed using distinct chips and media batches. Each media condition was assigned to a separate channel, from which ten positions were imaged. On average, 14 mother machine chambers per position were analyzed (see Video S1 for an example of mother machine microscopy data used to track single-cell growth over time).

Video S1. Example of single-cell growth in a mother machine used for growth-rate quantification

### Quantification and statistical analysis

#### Batch culture measurements and data analysis

Flasks were sampled hourly by collecting 0.5 mL using a sterile serological pipette. Samples were collected leaving the flasks in the water bath after stopping the shaking motion. OD_600_ and GFP fluorescence were measured using a plate reader (Synergy Mx, BioTek). GFP fluorescence was measured with excitation at 485/20.0 nm and emission at 535/20.0 nm; gain was set to 50, and optics to “bottom read mode.” Measurements were performed without lid to prevent interference from condensation due to the warm cultures.

While OD_600_ reflects total biomass, including both WT and TS lysogens, GFP fluorescence and cell count measurements specifically quantify the GFP-expressing WT subpopulation. Samples from batch cultures were diluted 1:100 during the first 3 h and 1:1000 at later time points in PBS, and 200-μL aliquots were analyzed using a CytoFLEX flow cytometer V-B-R series (Beckman Coulter International SA) equipped with three lasers, two of which were used (Blue, 50 mW; Violet, 80 mW) emitting at a fixed wavelength of 488 nm and 405 nm to determine WT cell counts. Measurements were performed at a pre-set flow rate of 60 μL min^−1^. An electronic gating strategy was applied to exclude instrument and sample background, and all data were processed using CytExpert software (Version 2.3.0.84). Instrument settings and gating parameters were kept constant across all samples to ensure consistency and comparability.

Raw data for OD_600_, GFP fluorescence, and WT cell counts were compiled from Excel files and manually curated into a unified dataset. All experiments were performed with four biological replicates, where each replicate represents independent cultures measured under identical conditions. The dataset was analyzed using custom scripts written in Python 3.10.4, which were used for data processing, statistical analysis, and generation of all the plots.

For all measurements, central tendency is reported as the mean, and variability is represented as the standard deviation (SD), as indicated in figure legends. No data points were excluded from the analysis. Statistical analyses were descriptive and aimed at quantifying trends and variability; no formal hypothesis testing was performed.

#### Automated image analysis with MIDAP

The acquired images were processed using a custom-made software called MIDAP (Version 0.3.18), developed by Franziska Oschmann, Janis Fluri, Lukas von Ziegler, and Thomas Wuest (https://github.com/Microbial-Systems-Ecology/midap). MIDAP allows the user to decide which region of the image needs to be further processed by manually selecting the area of the image. Segmentation was performed using the Omnipose algorithm (Version 0.4.4),^70^ implemented within MIDAP. Specifically, we used the *bact_fluor_omni* and *bact_phase_omni* models from MIDAP. These models were trained following the procedure described in the original Omnipose publication, using the publicly available training dataset. Briefly, a base model was trained from scratch with the following parameters: 4000 epochs, learning rate of 0.1, diameter set to 0, batch size of 16, and the RAdam optimizer. Subsequently, this base model was fine-tuned using in-house training data optimized for our image type with 50 additional epochs and identical training parameters. Tracking of individual cells over time was conducted using the STrack algorithm (Version 4),^71^ which links segmented objects across frames based on spatial proximity and object size consistency.

#### Single-cell growth rate measurements and statistical analysis

Single-cell growth rates were calculated based on cell elongation. MIDAP reports the length of the major axis, and assuming cells are growing exponentially, it is possible to calculate the slope of the regression line taken from the logarithmic-converted length.^79^ Only cells detected in at least 7 frames were kept for further processing. As cells proliferate within the chambers, those located near the main channel will exit the imaging field, leading to artifactual negative growth rates. We thus excluded cells whose x-coordinates exceeded a predefined threshold near the chamber exit. Finally, only cells with an *R*^2^ ≥ 0.95 from a simple linear regression (with intercept) were retained, where *R*^2^ reflects the squared Pearson correlation between growth rate and time, indicating linear fit quality. More details about the filtering can be found in Table S2.

Assuming a normal distribution of single-cell growth rates, a two-way ANOVA was performed to assess the effects of experimental conditions and biological replication. Post-hoc comparisons between conditions were conducted using Tukey’s Honestly Significant Difference (HSD) test, implemented via the pairwise *tukeyhsd* function from the *statsmodels* package in Python, to identify statistically significant differences and report adjusted *p*-values. Data processing, plotting, and statistical analysis were carried out using custom scripts developed in Python 3.10.4.

#### Consumer-resource model

The model is phenomenological and designed to identify parameter regimes consistent with the observed short-term population dynamics rather than to represent detailed intracellular regulation. We describe population dynamics using ordinary differential equations that track glucose (C_g_), lysate (C_l_), wild-type biomass (B_WT_), and temperature-sensitive biomass (B_TS_) partitioned into k sequential compartments (B_TS,1_, B_TS,2_, …, B_TS,k_). Total TS biomass is given by B_TS_ = Σ B_TS,i_. Optical density at 600 nm (OD_600_) represents the sum of live biomass (B_WT_ + B_TS_) where lysate does not directly contribute. All variables are expressed in biomass-equivalent units (yield Y = 1). Time is in hours, and growth rates are in h^−1^.

Growth depends on substrate concentration according to Monod kinetics:

(Equation 1) $$\mu_{y}\left(C_{y}\right)=\mu_{y,\max}\frac{C_{y}}{C_{y}+K_{S,y}}\;with\;y\in \left\{g,l\right\}$$

For glucose, *K*_*S,g*_ is small, such that *μ*_*g*_(C_g_) ≈ *μ*_*g,max*_ over the measured range. For lysate, concentrations are typically much lower than *K*_*S,l*_, resulting in an approximately linear dependence on C_l_. Nevertheless, the full Monod form was retained for both substrates to ensure consistency.

Glucose is consumed by both WT and TS cells:

(Equation 2) $$\frac{d}{dt}C_{g}=-\mu_{g}\left(C_{g}\right)\left(B_{WT}+B_{TS}\right)$$

Lysate is generated through lysis of the terminal TS compartment and consumed by both WT and TS cells:

(Equation 3) $$\frac{d}{dt}C_{l}=-\mu_{l}\left(C_{l}\right)\left(B_{WT}+B_{TS}\right)+\varepsilon k\delta B_{TS,k}$$

Lysate uptake is permitted for both WT and TS cells in the model, allowing TS cells to transiently consume lysate prior to lysis.

WT cells grow additively on both substrates:

(Equation 4) $$\frac{d}{dt}B_{WT}=\left[\mu_{g}\left(C_{g}\right)+\mu_{l}\;\left(C_{l}\right)\right]B_{WT}$$

Alternative co-limitation formulations are possible,^80^^,^^81^ but the available data do not allow us to distinguish between them. We therefore adopt the additive form as a simple phenomenological choice, representing lysate uptake as an additional growth contribution over the short time window studied, without strongly suppressing glucose-based growth.

Induction-to-lysis timing is represented as a linear-chain (Erlang) process, in which TS biomass progresses through k compartments. Cells in each compartment grow on glucose and advance at rate kλ, giving:

(Equation 5) $$\frac{d}{dt}B_{TS,1}=\mu_{g}\left(C_{g}\right)B_{TS,1}-k\delta B_{TS,1}$$

(Equation 6) $$\frac{d}{dt}B_{TS,i}=\mu_{g}\left(C_{g}\right)B_{TS,i}-k\delta B_{TS,i}+k\delta B_{TS,i-1}\;for\;i=2,.\;.\;.,n$$

Biomass exiting the last compartment of the chain, B_TS,k_, undergoes lysis, ceases glucose uptake, and contributes εkδB_TS,k_ to the lysate pool C_l_. This construction yields an Erlang(k, kδ) waiting-time distribution with mean 1/δ and variance 1/(kδ^2^), allowing both mean and dispersion of lysis times to be tuned.

Parameters are initialized as follows: at t = 0, B_WT_(0) = WT_i_, B_TS,1_(0) = TS_i_, B_TS,i>1_(0) = 0, and C_l_(0) = 0. Glucose is initialized to the experimentally measured concentration corresponding to the observed increase in biomass (B_WT,final_ - B_WT,inital_) or omitted in simulations where *μ*_*g*_ is fixed.

#### Model parametrization

Model parameters were fitted by nonlinear least squares (SciPy *least_squares*) using log-transformed OD_600_ residuals weighted by the replicate standard deviation. For each mix and time point, the residual was defined as:

(Equation 7) $$r=\frac{\log\left(ODmean\right)-\log\left(ODmodel\right)}{SD}$$

Model trajectories were interpolated to observation times, and very small standard deviation (SD) values were floored at 10^−3^ to stabilize the weighted least-squares fit and prevent disproportionate weighting of individual time points.

We performed the parametrization in three sequential stages.

• Stage 1: Glucose kinetics. We estimated the glucose growth parameters *μ*_*g,max*_ and *K*_*S,g*_ from the 100WT population.
• Stage 2: Lysis dynamics. We fitted the TS lysis rate (*δ*) and the number of lysis compartments (k) from the first four time points of the TS-only (100TS) population. Note that without lysis-time heterogeneity (k = 1), the model would reduce to instantaneous lysis, which is incompatible with the gradual decline observed in the experimental data. A compartmental structure (k > 1) is therefore required to reproduce the distributed lysis timing and match the early-time dynamics.
• Stage 3: Lysate dynamics. With parameters from Stages 1 and 2 fixed, we estimated parameter values for lysate-dependent growth (*μ*_*l*_, *K*_*S,l*_), and the biomass recycling efficiency (*ε*). Fits were based on the 73WT–27TS and 19WT–81TS mixtures, ensuring that only mixed populations informed lysate-related parameters.

Parameter bounds were imposed to avoid non-identifiable regions. Model diagnostics for goodness of fit included the residual sum of squares (RSS), the Akaike information criterion (AIC), and the Bayesian information criterion (BIC).

To evaluate the robustness of parameter estimates, we applied nonparametric bootstrapping across all three modeling stages. For each stage, experimental replicates were resampled with replacement, and the resampled datasets were re-fit using the same fitting procedure as for the original data. From the resulting distributions of best-fit parameters, 95% confidence intervals were derived as the 2.5th–97.5th percentiles. The lower and upper bounds of these intervals are reported in Table S1.
