# Supplementary material for: Phage-mediated lysis increases growth rate of surviving bacterial cells
Source: iScience. 2026 Apr 25;29(6):115899. doi: 10.1016/j.isci.2026.115899 (PMC13196028; doi:10.1016/j.isci.2026.115899)
Supplement: Document S1. Figures S1–S5 and Tables S1–S6 [file mmc1.pdf]

iScience, Volume *29*

Supplemental information

Phage-mediated lysis increases growth rate of surviving bacterial cells

Emanuele Fara, Benjamin Raach, Alessio Cavallaro, Justus Fink, Divvya Ramesh, Yongzhao Guo, Victoria Orphan, Alex R. Hall, Gabriele Micali, Martin Ackermann, and Olga T. Schubert

FIGURES

S1 WT-only biomass via cell count and GFP fluorescence
S2 Parameter sensitivity analysis of the 19WT–81TS model
S3 Dynamics of TS lysis, lysate production, and WT growth from the model
S4 Estimation of lysed-derived biomass and quantification of supernatant properties
S5 pEF plasmid designs

TABLES

S1 Best-fit model parameter values
S2 Selected cells by minimum frame threshold with growth rates measured from length
S3 ANOVA test results on growth rate across conditions
S4 Tukey's HSD post-hoc test for pairwise comparisons
S5 List of DNA fragments for pEF plasmids
S6 List of primers for plasmid sequencing

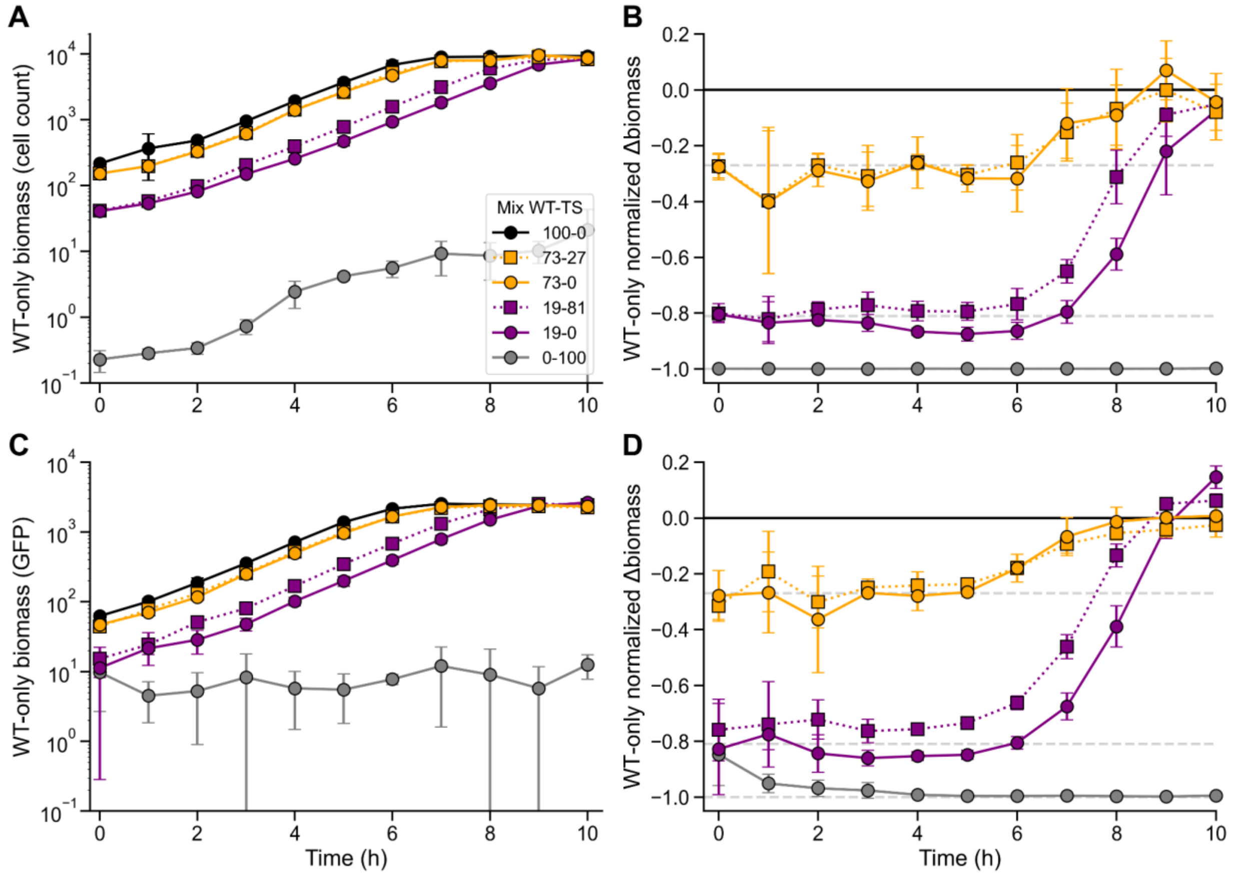

Figure S1. WT-only biomass via cell count and GFP fluorescence, Related to Figure 2.

(A) WT-only biomass over time in cell count of GFP-expressing WT lysogens measured via flow cytometry. Each data point represents the mean ± standard deviation (SD) from four independent biological replicates.

(B) Normalized WT-only biomass relative to the 100WT control. The 19WT–81TS population begins with the same WT cell count as the 19WT control but shows a sustained increase after lysis at 1 hour, consistent with partial compensation driven by WT cells.

(C) Growth curves of WT-only cultures measured by GFP fluorescence using a plate reader, serving as an independent proxy for WT-only biomass and corresponding to the cell count measurements obtained via flow cytometry shown in A.

(D) WT biomass normalized to the 100WT control, analogous to the normalization shown for WT cell count data in B.

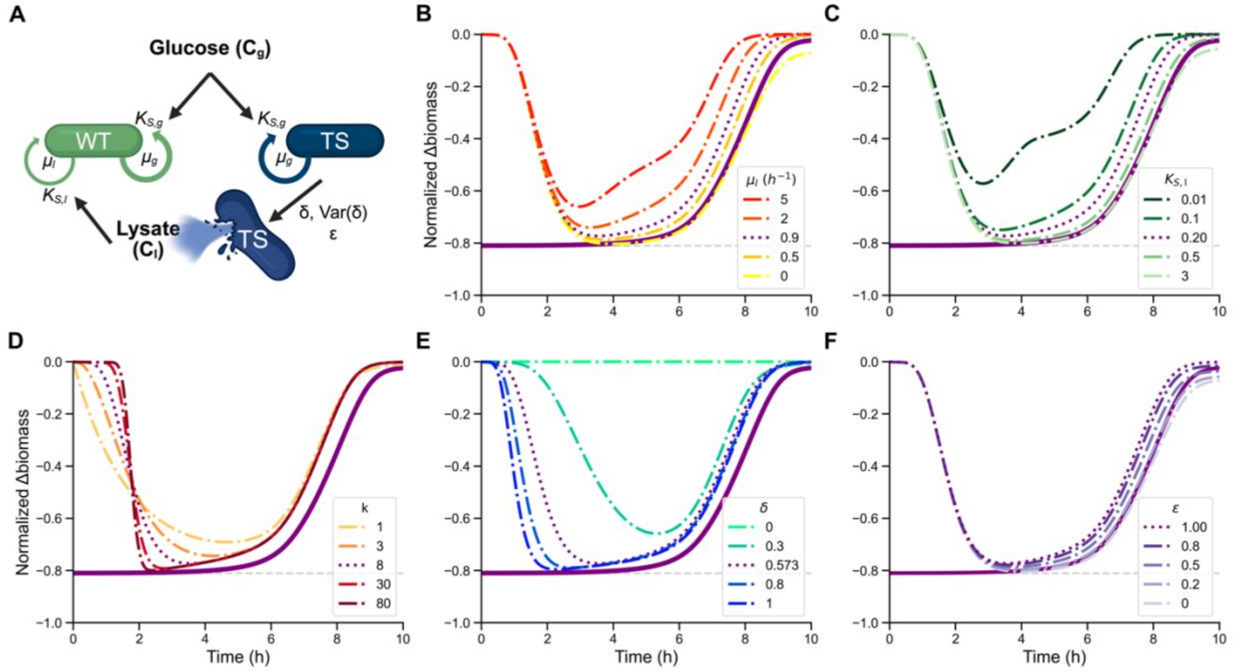

Figure S2. Parameter sensitivity analysis of the 19WT–91TS model, Related to Figure 3.

(A) Model schematic (same as Figure 3A) showing interactions among WT lysogens, TS lysogens, glucose, and lysate. Lysate represents inactive biomass produced by lysis; its uptake by surviving cells corresponds to a reactivation of biomass. The net contribution of recycling to community recovery depends both on the recycling efficiency (ϵ) and on the recycling speed governed by μ_l and $K_{S,l}$. Across all panels, the solid purple line represents the 19WT control population, whereas the dotted purple line corresponds to the 19WT–81TS population simulated with the best-fit parameters.

(B) Effect of maximum TS growth rate on lysate, μ_l. Higher μ_l accelerates lysate consumption by fast biomass reactivation.

(C) Effect of the Monod constant for lysate, $K_{S,l}$. A lower $K_{S,l}$ means higher affinity for lysate components and thus increased growth at low lysate concentrations and enhanced compensation. $K_{S,l}$ modulates the effective growth rate similarly to μ_l, but with lower $K_{S,l}$, recycling becomes faster at low lysate levels. The best-fit parameter set corresponds to low $K_{S,l}$.

(D) Effect of the number of compartments, k. Increasing the number of compartments leads to more synchronized lysis events, which sharpens the biomass decline but does not substantially alter the timing or magnitude of the subsequent recovery.

(E) Effect of lysis rate, δ. Varying the lysis rate also affects population dynamics, with the extremes defined by $\delta = 0$, where no lysis occurs, and $\delta = 1$, where lysis is immediate.

(F) Effect of biomass recycling efficiency, ε. Higher ε increases the fraction of lysed biomass that is reusable, thereby strengthening WT recovery.

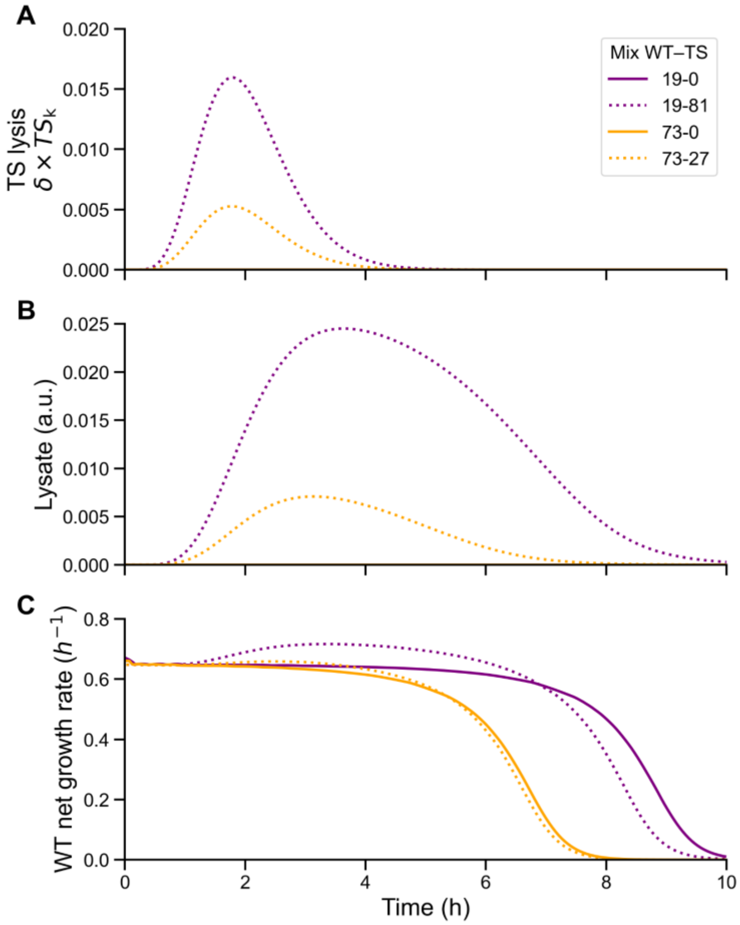

Figure S3. Dynamics of TS lysis, lysate production, and WT growth from the model, Related to Figure 3.

(A) TS cells undergoing lysis, estimated as the biomass exiting the last compartment of the TS population (δ x TS_k).

(B) Concentration of consumable lysate according to the model. The dynamic is a consequence of released lysate upon TS lysis and subsequently consumed by WT and TS cells.

(C) Net growth rate of the WT subpopulation (as in Figure 3D), illustrating the transient increase in WT growth that coincides with lysate release and uptake. Together, these panels illustrate how the timing of lysis and the speed of lysate consumption determine the magnitude of biomass recovery. In the model, the recycling efficiency contributes to compensation only because the released material is taken up rapidly, demonstrating that fast recycling is essential for the observed short-term growth advantage of surviving WT cells.

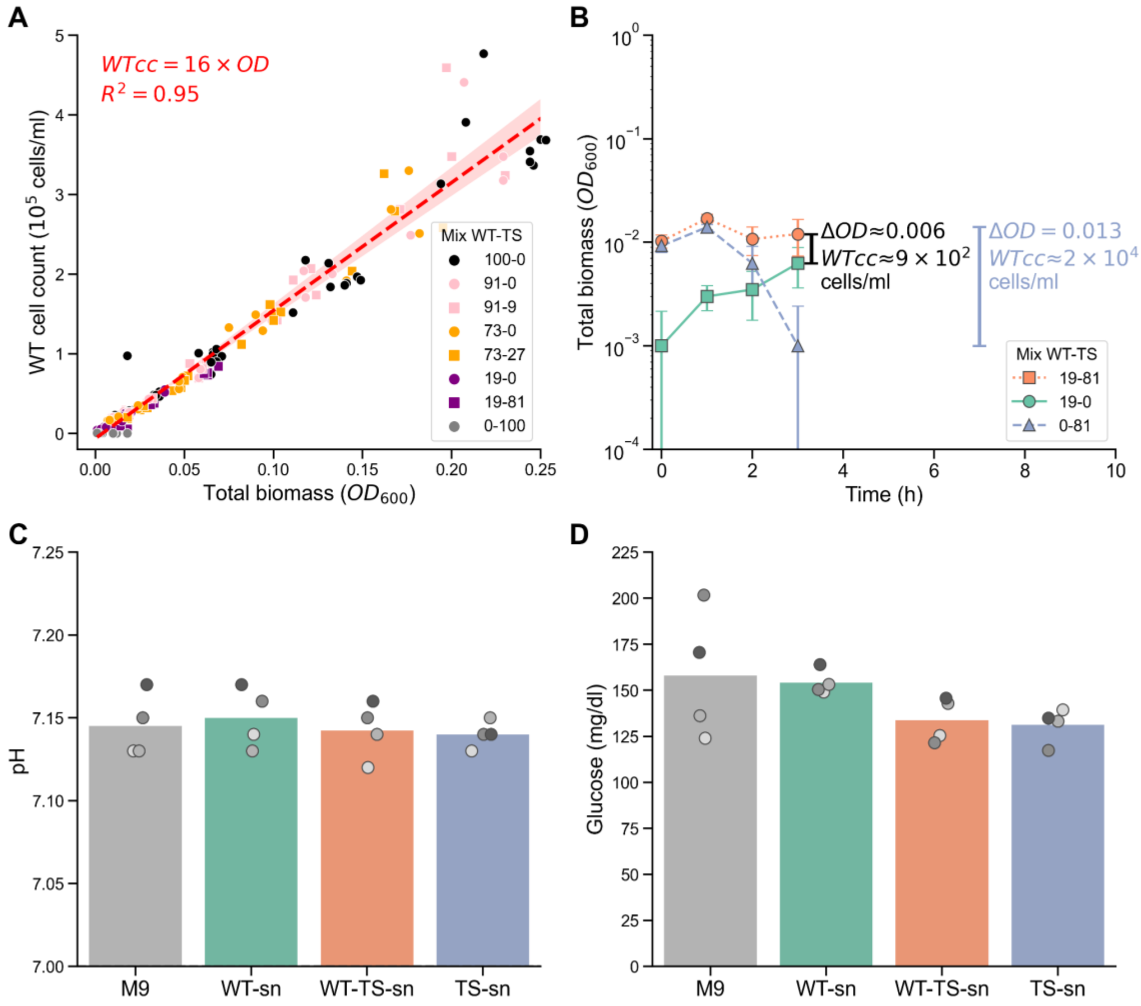

Figure S4. Estimation of lysed-derived biomass and quantification of supernatant properties, Related to Figure 4.

(A) Correlation between WT cell counts measured by flow cytometry and OD_{600} across flask samples, used to derive the OD-to-cell-number conversion factor. Data points represent four biological replicates for each WT–TS mixture.

(B) Batch-culture growth curves during the first 3 hours of incubation, corresponding to the period before supernatant collection. The estimated number of lysed TS cells was calculated from the ΔOD in the 81TS culture between 1 and 3 hours using the conversion factor in panel A. The ΔOD at 3 hours between the 19WT–81TS co-culture and the 19WT monoculture reflects the net biomass gained by WT cells due to TS lysis. Comparing biomass lost and gained indicates that ~22 lysed cells support the growth of one new WT cell. Each data point represents the mean ± standard deviation (SD) from four biological replicates.

(C) pH values of all supernatants used in the microfluidic experiments, including unconditioned M9 medium. Each circle denotes a biological replicate, shaded as in Figure 4B.

(D) Glucose concentrations of the same supernatants; slightly lower values in WT–TS-sn and TS-sn remained well above levels expected to limit growth.

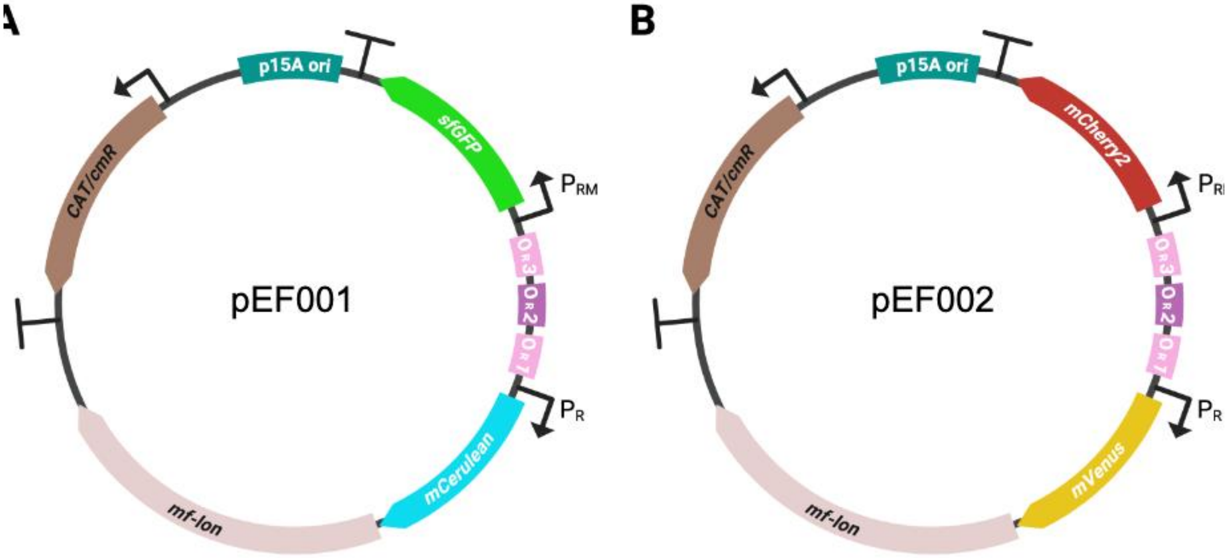

Figure S5. pEF plasmid designs, Related to STAR Methods.

Schematic representation of the two expression plasmids pEF001 (A) and pEF002 (B) used in this study to label the WT and TS lysogens with fluorescent proteins. Both plasmids are based on a p15A origin of replication and carry a chloramphenicol resistance cassette (*CmR*) for plasmid maintenance. In the lysogenic state, the promoter P_{RM}, which is active when the CI repressor is bound to operator sites O_R1 and O_R2, drives expression of the lysogenic reporter gene – either *sfGFP* (GFP) in pEF001 or *mCherry2* (RFP) in pEF002. Upon temperature-induced inactivation or dissociation of CI from operator site O_R3, repression of the lytic promoter P_R is relieved. This activates the transcription of genes associated with the lytic cycle and, on the plasmids, triggers expression of the lytic reporter – *mCerulean* (CFP) in pEF001 or *mVenus* (YFP) in pEF002. This regulatory architecture enables state-specific fluorescent labeling: green or red fluorescence during lysogeny, and cyan or yellow fluorescence during lysis. Plasmid sequences are available in GenBank, see STAR Methods.

Parameter	Description	Parametrization	Best-fit value	Bootstrap 95% CI lower	Bootstrap 95% CI upper
μ_g	Growth rate on glucose	Stage 1	0.7848 h^{-1}	0.6263	0.9927
μ_l	Growth rate on lysate	Stage 3	0.9109 h^{-1}	0.8276	1.849
$K_{S,g}$	Monod constant for glucose	Stage 1	0.0878	0.001	0.2
$K_{S,l}$	Monod constant for lysate	Stage 3	0.2	0.2	0.2
δ	TS lysis rate	Stage 2	0.5649 h^{-1}	0.5570	0.5887
k	TS compartments	Stage 2	8	6	10
ε	Biomass recycling efficiency	Stage 3	99.99 (%)	66.53	100

Table S1. Best-fit model parameter values, Related to Figure 3.
Parameter values were estimated by fitting a consumer-resource model to experimental growth data in three sequential stages, minimizing log-scale residuals between model predictions and observations. Uncertainty is shown as bootstrap 95% CIs, whose lower/upper bounds are the empirical 2.5th and 97.5th percentiles of parameter estimates obtained by resampling experimental replicates with replacement (e.g., B = 500 bootstrap datasets). Best-fit values are the point estimates that minimize the objective.

Min # frames	Selected by frame	Selected by R^2 length	% selected cells	Discarded cells	Max growth rate	Min growth rate
3	42,381	36,458	42.8	48,755	1.44	-9.03
4	39,503	34,597	40.6	50,616	1.31	-6.21
5	37,252	33,020	38.8	52,193	1.30	-5.07
6	35,324	31,549	37.0	53,664	1.12	-2.29
**7**	**33,515**	**30,091**	**35.3**	**55,122**	**1.00**	**0.06**
8	31,764	28,618	33.6	56,595	0.98	0.06
9	30,261	27,334	32.1	57,879	0.98	0.06
10	28,624	25,894	30.4	59,319	0.98	0.06

Table S2. Selected cells by minimum frame threshold with growth rates measured from length, Related to Figure 4.

Summary of growth rate measurements for cells filtered by spatial and temporal criteria. From an initial population of 85,213 cells, filtering based on x-coordinate reduced the dataset to 52,698 cells. Further filtering was achieved by screening for a minimum number of frames (ranging from 3 to 10) and selecting only those cells with an R^2 value exceeding 0.95, thereby ensuring the analysis of cells exhibiting consistent exponential growth. In this table, the major axis length was used to calculate both the growth rate and its corresponding R^2 value. A frame filter of 7 was chosen for subsequent analyses because it provided the optimal balance between retaining enough cells and eliminating negative growth rates. This filter ensures that cells are tracked for at least 30 minutes (time between 7 frames). Importantly, this duration should not exclude fast-growing cells, as it is very unlikely that any cell under the tested conditions (*E. coli* in M9 + glucose, with a doubling time of 42 ± 12 minutes) would have a shorter doubling time.

Effect	Sum of squares	df	F	p-value
C(Condition)	0.0169	2	56.3019	**0.0001 (***)**
C(Replicate)	0.0075	3	16.7732	**0.0025**
Residual	0.0009	6	–	–

Table S3. ANOVA test results on growth rate across conditions, Related to Figure 4.
Two-way ANOVA testing effects of condition and replicate identity on single-cell growth rate. Reported values include sum of squares, degrees of freedom, F-statistic, and p-value.

Group 1	Group 2	Mean diff.	p-adj	Lower	Upper	Reject
WT-sn	TS-sn	0.0862	**0.0081 (**)**	0.0257	0.1467	True
WT-sn	WT–TS-sn	0.0708	**0.0239 (*)**	0.0103	0.1313	True
TS-sn	WT–TS-sn	-0.0154	**0.7642**	-0.0759	0.0451	False

Table S4. Tukey's HSD post-hoc test for pairwise comparisons, Related to Figure 4.
Post-hoc analysis following ANOVA, showing mean differences between groups, adjusted p-values, 95% confidence intervals, and whether the difference is statistically significant. P-values shown in bold correspond to the values called out in Figure 4B.

DNA fragment	Type	Plasmid pEF00X	Source plasmid	Resource	Reference
p15A	Origin of replication	1, 2	PRM-GFP	Addgene #40127	Huang et al.[S1]
CAT/cmR	Antibiotic resistance	1, 2	pBAD33	GenBank: LC760188.1	–
mf-lon	Lon protease	1, 2	–	GenBank: KM521209	Cameron et al.[S2]
pdt #3	Degradation tag	1, 2	–	–	Cameron et al.[S2]
OR	Lambda switch	1, 2	pJPC12	Addgene #80859	Brödel et al.[S3]
lambda t0	Terminator	1, 2	–	iGEM: K3257021	–
T7Te	Terminator	1, 2	PRM-GFP	Addgene #40127	Huang et al.[S1]
BBa_B0014	Bidirectional terminator	1, 2	–	iGEM: B0014	–
sfGFP	Lysogenic reporter	1	pJPC12	Addgene #80859	Brödel et al.[S3] Pédelacq et al.[S4]
mCerulean	Lytic reporter	1	pEB1-mCerulean	Addgene #103968	Rizzo et al.[S5] Belleza et al.[S6]
mCherry2	Lysogenic reporter	2	mCherry-pBAD	Addgene #54630	Shen et al.[S7] Shaner et al.[S8]
mVenus	Lytic reporter	2	pEB1-mVenus	Addgene #103986	Belleza et al.[S6] Kremers et al.[S9]

Table S5. List of DNA fragments for pEF plasmids, Related to STAR Methods.
Each DNA fragment was sourced from plasmids available in Addgene, GenBank, or the iGEM repository. The sequences between the fragments were also derived from these sources. The nucleotide sequences of the fluorescent proteins were codon-optimized for *Escherichia coli* using the Codon Optimization Tool provided by Integrated DNA Technologies (IDT) during the gBlock ordering process. This optimization ensures optimal protein expression in the host strain.

Name	Sequence (5' – 3")	Target	Plasmid pEF00X
p15A_1	TCAAATCAGTGGTGGCGAAAC	p15A	1, 2
GFP_2	ACCAACGGTAAGCTGACCTTG	GFP	1
RFP_2	TCCTTTTGCTTGGGACATCCTG	GFP	2
GFP_3	TCCAGCAGCACCATGTGATC	RFP	1
RFP_3	TTGACCTCGGCATCGTAATGAC	RFP	2
CFP_4	AGCAGCGGTAACGAACTCAAG	CFP	1
YFP_4	GTAGGACAGGTAATGGTTGTCTGG	YFP	2
CFP_5	ACCCAGACCACATGAAACAGC	CFP	1
YFP_5	ACACTTGTCACTACTTTGGGTTATG	YFP	2
mf-lon_6	TTCAATAATGGACCAGCGAGTCTTC	mf-lon	1, 2
mf-lon_7	ATGCTGAAGTCGAGTTGATCGAG	mf-lon	1, 2
mf-lon_8	CTTTCTTGTCTACGCCAAACTTCTC	mf-lon	1, 2
mf-lon_9	AACGTATCTTCGACCATACCGAG	mf-lon	1, 2
cmR_10	GTGAGCTGGTGATATGGGATAGTG	cmR	1, 2
cmR_11	CATGATGAACCTGAATCGCCAG	cmR	1, 2
p15A_12	AAATCAATTACCAGTGGCTGCTG	p15A	1, 2

Table S6. List of primers for plasmid sequencing, Related to STAR Methods.
Primers were designed in alternating orientations to ensure complete coverage of each plasmid sequence. They were also used in colony PCR to confirm successful Gibson assembly. For example, primers p15A_1 and GFP_2 amplify a region of approximately 1500 bp spanning three of the five fragments.

SUPPLEMENTAL REFERENCES

[S1]. Huang, D., Holtz, W.J., and Maharbiz, M.M. (2012). A genetic bistable switch utilizing nonlinear protein degradation. *J. Biol. Eng.* 6, 9. https://doi.org/10.1186/1754-1611-6-9

[S2]. Cameron, D.E., and Collins, J.J. (2014). Tunable protein degradation in bacteria. *Nat. Biotechnol.* 32, 1276–1281. https://doi.org/10.1038/nbt.3053

[S3]. Brödel, A.K., Jaramillo, A., and Isalan, M. (2016). Engineering orthogonal dual transcription factors for multi-input synthetic promoters. *Nat. Commun.* 7, 13858. https://doi.org/10.1038/ncomms13858

[S4]. Pédelacq, J.-D., Cabantous, S., Tran, T., Terwilliger, T.C., and Waldo, G.S. (2006). Engineering and characterization of a superfolder green fluorescent protein. *Nat. Biotechnol.* 24, 79–88. https://doi.org/10.1038/nbt1172

[S5]. Rizzo, M.A., and Piston, D.W. (2005). High-Contrast Imaging of Fluorescent Protein FRET by Fluorescence Polarization Microscopy. *Biophys. J.* 88, L14–L16. https://doi.org/10.1529/biophysj.104.055442

[S6]. Balleza, E., Kim, J.M., and Cluzel, P. (2018). Systematic characterization of maturation time of fluorescent proteins in living cells. *Nat. Methods* 15, 47–51. https://doi.org/10.1038/nmeth.4509

[S7]. Shen, Y., Chen, Y., Wu, J., Shaner, N.C., and Campbell, R.E. (2017). Engineering of mCherry variants with long Stokes shift, red-shifted fluorescence, and low cytotoxicity. *PLoS ONE* 12(2): e0171257. https://doi.org/10.1371/journal.pone.0171257

[S8]. Shaner, N.C., Campbell, R.E., Steinbach, P.A., Giepmans, B.N.G., Palmer, A.E., and Tsien, R.Y. (2004). Improved monomeric red, orange and yellow fluorescent proteins derived from *Discosoma* sp. red fluorescent protein. *Nat. Biotechnol.* 22, 1567–1572. https://doi.org/10.1038/nbt1037

[S9]. Kremers, G.-J., Goedhart, J., van Munster, E.B., and Gadella, T.W.J. (2006). Cyan and Yellow Super Fluorescent Proteins with Improved Brightness, Protein Folding, and FRET Förster Radius. *Biochemistry* 45, 6570–6580. https://doi.org/10.1021/bi0516273
